# Stimulation of TLR4 by recombinant HSP70 requires structural integrity of the HSP70 protein itself

**DOI:** 10.1186/1476-9255-9-11

**Published:** 2012-03-26

**Authors:** Michael Luong, Yanyu Zhang, Tim Chamberlain, Tianhui Zhou, Jill F Wright, Ken Dower, J Perry Hall

**Affiliations:** 1 Inflammation and Remodeling Research Unit, Pfizer, Cambridge, Massachusetts, USA; 2 Immunology and Autoimmunity Research Unit, Pfizer, Cambridge, Massachusetts, USA; 3 BioTherapeutics Clinical Program, Pfizer, Collegeville, Pennsylvania, USA

**Keywords:** Endotoxin, Heat shock protein 70, Polymyxin B, Proteinase K, TLR4

## Abstract

**Background:**

Toll-like receptor 4 (TLR4) is activated by bacterial endotoxin, a prototypical pathogen-associated molecular pattern (PAMP). It has been suggested that TLR4 can also be activated by damage-associated molecular pattern (DAMP) proteins such as HSP70. It remains a challenge to provide unequivocal evidence that DAMP proteins themselves play a role in TLR4 activation, as the DAMP proteins used are often contaminated with endotoxin and other TLR ligands introduced during protein expression and/or purification.

**Results:**

Here we report that the activation of TLR4 on primary human macrophage cultures by recombinant HSP70 is not solely due to contaminating endotoxin. Polymyxin B pretreatment of HSP70 preparations to neutralize contaminating endotoxin caused significant reductions in the amount of TNF-α induced by the recombinant protein as determined by ELISA. However, digestion of HSP70 with Proteinase K-agarose beads also dramatically reduced the TNF-α response of macrophages to HSP70, while leaving levels of contaminating endotoxin largely unchanged relative to controls.

**Conclusions:**

These results indicate that the stimulatory effect of recombinant HSP70 requires both the presence of endotoxin and structural integrity of the heat shock protein itself.

## Introduction

TLRs are activated by PAMPs contained in lipids, lipopeptides, proteins, and nucleic acids of microbial origin [1]. There is increasing evidence that TLRs can also be activated by host-derived molecules released from sites of tissue damage, collectively known as damage-associated molecular patterns (DAMPs) [2]. These putative DAMPs can be proteins that are released from necrotic cells or proteins that are expressed in response to inflammatory stimuli. It has been suggested that heat shock protein 70 (HSP70) may function as a DAMP for TLR4 and other receptors (e.g., see [3,4]). However, obtaining proof that HSP70 acts as a DAMP for TLR4 is challenging given that preparations of purified recombinant HSP70 are often contaminated with PAMPs. Indeed, it has been reported that the activation of cultured macrophages by recombinant human HSP70 is the result of contaminating endotoxin [5].

We were interested in examining the activation of TLR4 on cultured human macrophages by preparations of commercially-available, low-endotoxin recombinant human HSP70. To evaluate the role of contaminating endotoxin in these preparations, we initially employed a traditional approach using Polymyxin B treatment [6]. We demonstrate here that Polymyxin B treatment to neutralize contaminating endotoxin caused significant reductions in the stimulatory effect of recombinant HSP70. Subsequently, we refined a protease digestion technique that had been suggested in the literature [7-10]. Proteinase K-agarose digestion of recombinant HSP70 dramatically lowered the stimulatory capacity of this protein preparation on human macrophages, while leaving levels of contaminating endotoxin unchanged relative to appropriate controls. These results support to the idea that the HSP70 protein can function in concert with endotoxin to cause TLR4 activation.

## Methods

### Reagents

Recombinant low-endotoxin human HSP70 (cat. # ADI-ESP-555-F; lot # 05040922) and ultrapure LPSs (cat. # ALX-581-007-L002 and ALX-581-013-L002) were from Enzo Life Sciences (Plymouth Meeting, PA). The *Limulus *amoebocyte lysate assay was from Charles River Laboratories (Wilmington, MA). Polymyxin B solution, and Proteinase K-agarose from Tritirachium album, were from Sigma (St. Louis, MO). Agarose for control digestions was from Thermo Scientific (Rockford, IL).

### Primary human macrophages and cytokine analyses

Monocytes were prepared from human blood buffy coats as described [11]. Macrophages were differentiated essentially as described [12]. Monocytes were plated on 96-well tissue culture plates at 100,000 cells in 0.2 mL per well of RPMI 1640 supplemented with 5% FBS, 10 units/mL penicillin, 10 μg/mL streptomycin, and 100 ng/mL recombinant human M-CSF (Millipore). Macrophages were differentiated over a 6 day culture period at 37°C and 5% CO_2_. Cell culture supernatants were analyzed for cytokine content by electrochemiluminescence detection on a Sector6000 plate reader according to the manufacturer's instructions (Meso Scale Discovery, Gaithersburg, MD).

### Protein digestion

Proteinase K-agarose was reconstituted in endotoxin-free water to 10 mg/mL, incubated at 4°C for 2 hr, and washed five times with endotoxin-free water. Control agarose was washed five times with endotoxin free water. Digestion buffer was prepared by supplementing PBS with 2.7 mM KCl, 1.5 mM K_2_PO_4_, 137 mM NaCl, and 8.1 mM Na_2_PO_4_. This buffer was used to wash the Proteinase K-agarose and control agarose an additional four times. Following the final wash, digestion buffer was removed to leave a 50:50 slurry of beads plus residual digestion buffer. 200 μg of HSP70 was incubated with 200 μL digestion buffer, control agarose slurry, or Proteinase K-agarose slurry on a shaking platform for 3 hr at 37°C. Following incubation, the upper, aqueous phases from protein samples treated with control agarose slurry or Proteinase K-agarose slurry were moved to fresh tubes. The concentrations of these samples were not significantly different as measured by OD280. Digestion was visualized by silver staining using the Invitrogen SilverQuest™ Silver Staining Kit (cat. # LC6070) according to the manufacturer's instructions.

### Statistical analyses

Statistical analyses were performed using the Prism software from GraphPad (La Jolla, CA). All data were analyzed using either one-way ANOVA with Tukey's post hoc tests, or two way ANOVA and repeated measure ANOVA with the Bonferroni post hoc tests for comparisons between groups. (*p < 0.05, **p < 0.005, and ***p < 0.0005).

## Results and discussion

**Figure 1:**
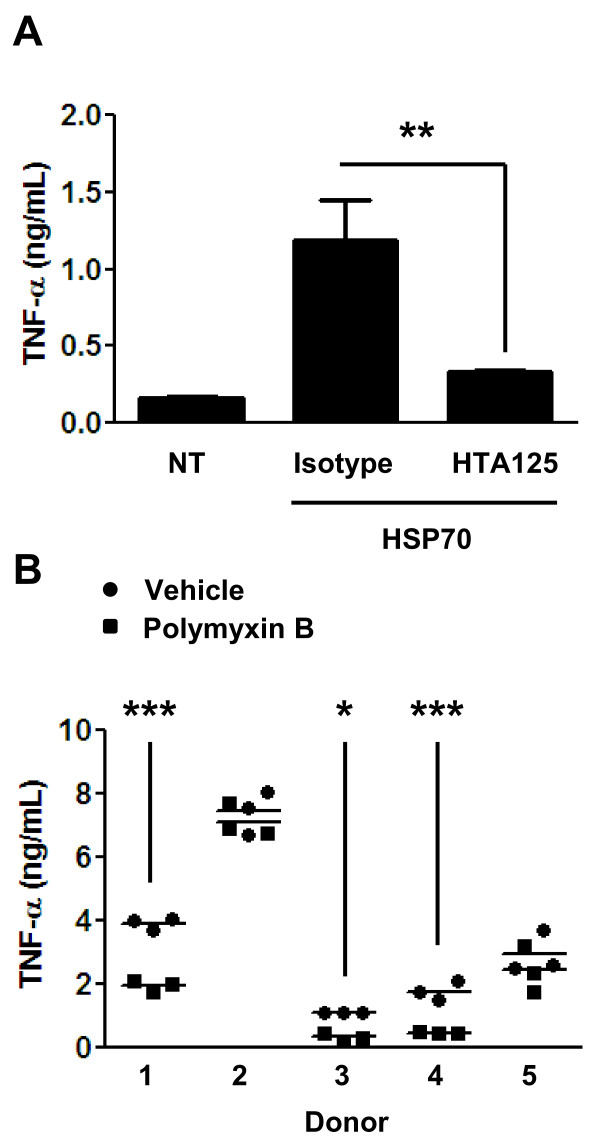
**The stimulatory effect of recombinant HSP70 on primary human macrophages is reduced by inhibition of TLR4, or by Polymyxin B treatment of the HSP70**. (A) Primary human macrophages were left untreated (NT), or treated with 100 μg/mL of HTA125 or an isotype control antibody for 30 min. Macrophages treated with antibodies were then stimulated for 4 hr with rhHSP70 at a final concentration of 10 μg/mL. Cell culture supernatants were collected and analyzed for TNF-α. **p < 0.005. (B) 5 μg rhHSP70 was pretreated with buffer alone or 10 μg Polymyxin B (at 100 μg/mL) for 30 min on ice, and then used to stimulate primary human macrophages for 4 hr at 10 μg/mL rhHSP70 final concentration in each culture. Cell culture supernatants were collected and analyzed for TNF-α. *p < 0.05, ***p < 0.0005.

We determined that commercially-available, recombinant, full-length human HSP70 induced pro-TNF-α production in cultures of primary human macrophages, and that these responses were blocked with an antagonistic anti-TLR4 antibody (Figure 1A). These results confirmed a role for TLR4 signaling in the induction of TNF-α production by the HSP70 preparation. However, they did not address whether this TLR4-dependent response is caused by the HSP70 protein itself, or any contaminating endotoxin that is present in the HSP70 preparation, or both. The recombinant HSP70 protein we used to stimulate macrophages is produced in *E. coli*; therefore, we routinely use the *Limulus *amoebocyte lysate assay to measure the endotoxin content. We found that these HSP70 preparations contained ≤ 6 EU/mg. We considered this level of contamination to be low, because we calculated that under the conditions used in our studies only 0.06 EU/mL of endotoxin would be introduced into our macrophage cultures by the addition of HSP70 to 10 μg/mL. We tested the ability of various forms of ultrapure LPS at 0.06 EU/mL to induce TNF-α production over a 4 hr period in primary human macrophages. We did not observe consistent and robust cytokine production in macrophage cultures under these conditions (data not shown). Nonetheless, to address the potential effect of any contaminating endotoxin, we sought to neutralize endotoxin in the HSP70 preparation with Polymyxin B prior to using it to stimulate macrophages. HSP70 from a single lot was pretreated with Polymyxin B, and then used to stimulate macrophages from five different donors for 4 hr. As shown in Figure 1B, in three out of the five macrophage isolates pre-treatment of the HSP70 with Polymyxin B caused significant reductions in HSP70 signaling activity, thus confirming a role for endotoxin in the stimulatory effect of this HSP70 preparation. As the reductions in HSP70 stimulatory activity were only observed in macrophage isolates from three out of five donors, we reasoned that this method of inactivating endotoxin with Polymyxin B can yield variable results and that some caution is required in their interpretation.

**Figure 2:**
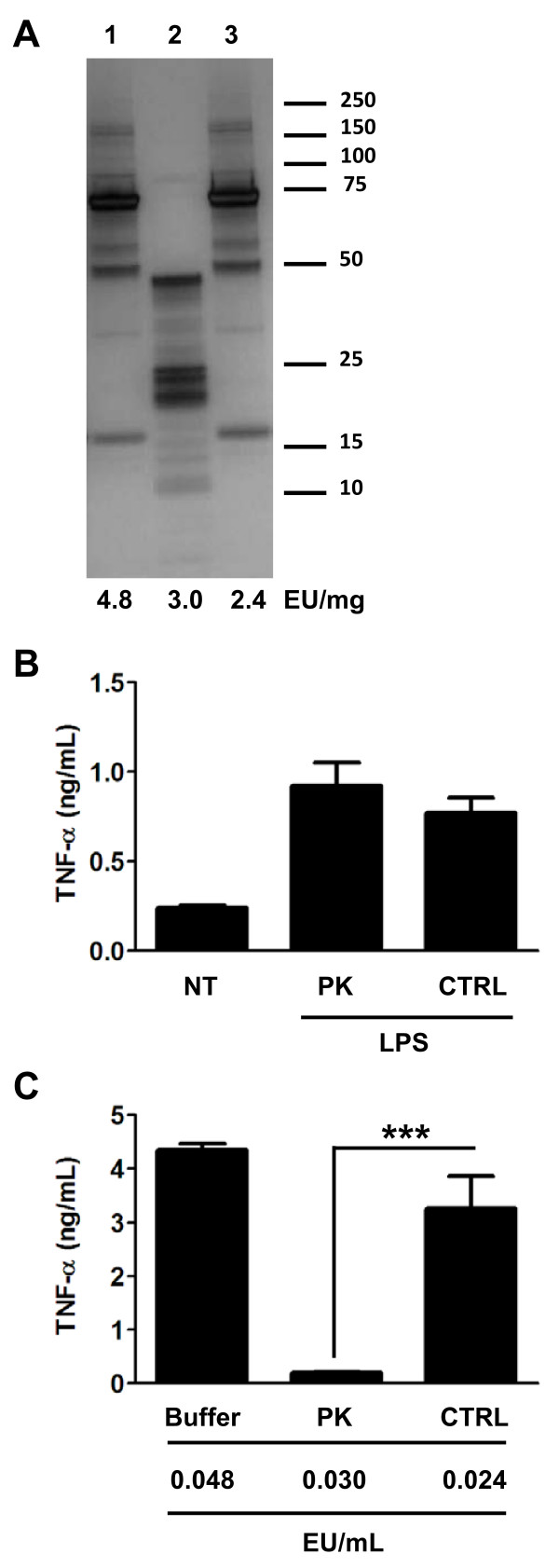
**Proteinase K-agarose digestion ablates HSP70 activity**. (A) 200 μg rhHSP70 was incubated with digestion buffer (lane 1), Proteinase K-agarose beads (lane 2), or control agarose beads (lane 3) for 3 hr at 37°C, as described in *Methods*. Protein integrity and endotoxin content were determined by silver staining and the LAL assay, respectively. (B) 100 pg of ultrapure LPS, which is an approximation of the endotoxin content in 200 μg of rhHSP70 (see *Results and Discussion*), was incubated with Proteinase K-agarose beads or control agarose beads, under the conditions described in *A*. The ultrapure LPS treated with Proteinase K-agarose beads (PK) or control agarose beads (CTRL) were then used to stimulate primary human macrophages for 16 hr at 100 pg/mL LPS final concentration in each culture. Cell culture supernatants were collected and analyzed for TNF-α. (C) The HSP70 preparations in *A *were used to stimulate primary human macrophages for 4 hr at 10 μg/mL final concentration. Cell culture supernatants were collected and analyzed for TNF-α. Endotoxin levels shown at bottom are the calculated amounts in the macrophage cultures based on the LAL determinations in *A*. Untreated macrophage cultures produced ≤ 100 pg/mL of TNF-α under these conditions. Results shown are representative of two independent digestion and stimulation experiments. ***p < 0.0005.

To determine whether the protein component of the HSP70 preparation played any role in the stimulation of macrophages, we evaluated and refined a technique that has been suggested in the literature and involves the use of proteases to disrupt protein integrity while leaving contaminating endotoxin intact [7-10]. We used Proteinase K-agarose beads to digest HSP70 at 37°C for 3 hr, and as controls the HSP70 preparation was treated with digestion buffer or control agarose beads under identical conditions. The silver stain gel in Figure 2A shows that the fully intact 70 kD HSP70 protein was digested by the Proteinase K-agarose treatment, and that under the digestion conditions employed none of the 70 kD protein remained. We employed Proteinase K treatment at 37°C because we had observed a complete reduction in the stimulatory capacity of ultrapure LPS following incubation at 55°C for 1 hr (not shown).

The use of Proteinase K-agarose beads facilitated separation of the recombinant protein component of the digest from the Proteinase K component of the digest, mitigating any potential effects of the Proteinase K enzyme on macrophages treated with the digested material. Indeed, in control experiments, macrophages treated with Proteinase K-agarose beads, or with soluble Proteinase K, prior to stimulation with ultrapure LPS, produced significantly less TNF-α compared to macrophages treated with control agarose beads, or vehicle, respectively (not shown). We also confirmed that the cell-stimulating activity of ultrapure LPS treated with Proteinase K-agarose beads was not reduced when compared with LPS treated with control agarose beads (Figure 2B).

Following Proteinase K-agarose or control agarose treatments the HSP70 preparations were used to stimulate macrophages. The cell-stimulating capacity of the HSP70 sample treated with Proteinase K-agarose beads was almost completely ablated relative to the HSP70 subjected to control agarose (Figure 2C). We also observed that treatment of HSP70 with the control agarose beads alone caused a substantial reduction in the level of endotoxin contamination of the HSP70 preparation compared to the buffer-alone control sample (Figure 2A). We speculate that the agarose beads may bind nonspecifically to endotoxin. Nevertheless, the endotoxin content of HSP70 treated with Proteinase K-agarose or control agarose beads were not dramatically different, with an even greater amount of endotoxin observed in the HSP70 subjected to Proteinase K-agarose compared to the amount of endotoxin observed in the HSP70 subjected to control agarose (Figure 2A). These results strongly implicate a role for the protein component in the stimulatory effect of these recombinant HSP70 preparations. Moreover, they have allowed us to speculate that protein structural motifs found only in fully intact HSP70 are required for TLR4 activation, be it through direct interaction with the TLR4 receptor complex, or through its ability to facilitate the transfer of bound endotoxin to MD2 on the TLR4 receptor complex, or through a mechanism that involves sensitization of the TLR4 receptor complex to levels of endotoxin that would normally be below levels required to induce robust signaling.

## Competing interests

The authors declare that they have no competing interests.

## Authors' contributions

**ML: **conceived and performed experiments, analyzed data, and helped write the manuscript. **YZ: **conceived and performed experiments, analyzed data, and helped write the manuscript. **TC**: conceived and performed experiments, analyzed data, and helped write the manuscript. **TZ: **analyzed data and helped write the manuscript. **JW: **conceived experiments, analyzed data, and helped write the manuscript. **KD: **conceived and performed experiments, analyzed data, and helped write the manuscript. **JPH: **conceived and performed experiments, analyzed data, and helped write the manuscript. All authors read and approved the final manuscript.
